# Differential Profiles of Gut Microbiota and Metabolites Associated with Host Shift of *Plutella xylostella*

**DOI:** 10.3390/ijms21176283

**Published:** 2020-08-30

**Authors:** Fei-Ying Yang, Hafiz Sohaib Ahmed Saqib, Jun-Hui Chen, Qian-Qian Ruan, Liette Vasseur, Wei-Yi He, Min-Sheng You

**Affiliations:** 1 State Key Laboratory for Ecological Pest Control of Fujian and Taiwan Crops, Institute of Applied Ecology, Fujian Agriculture and Forestry University, Fuzhou 350002, China; 18059141865@163.com (F.-Y.Y.); sohaibsaqib@gmail.com (H.S.A.S.); allenchen0426@gmail.com (J.-H.C.); 18258878869@163.com (Q.-Q.R.); lvasseur@brocku.ca (L.V.); 2 International Joint Research Laboratory of Ecological Pest Control, Ministry of Education, Fujian Agriculture and Forestry University, Fuzhou 350002, China; 3 Key Laboratory of Integrated Pest Management for Fujian-Taiwan Crops, Ministry of Agriculture, Fuzhou 350002, China; 4 Department of Biological Sciences, Faculty/School, Brock University, St. Catharines, ON L2S 3A1, Canada

**Keywords:** diamondback moth, insect–plant interaction, insect–microbe interaction, host expansion, metabolome, inter-omic

## Abstract

Evolutionary and ecological forces are important factors that shape gut microbial profiles in hosts, which can help insects adapt to different environments through modulating their metabolites. However, little is known about how gut microbes and metabolites are altered when lepidopteran pest species switch hosts. In the present study, using 16S-rDNA sequencing and mass spectrometry-based metabolomics, we analyzed the gut microbiota and metabolites of three populations of *Plutella xylostella*: one feeding on radish (PxR) and two feeding on peas (PxP; with PxP-1 and PxP-17 being the first and 17th generations after host shift from radish to peas, respectively). We found that the diversity of gut microbes in PxP-17 was significantly lower than those in PxR and PxP-1, which indicates a distinct change in gut microbiota after host shift. Kyoto Encyclopedia of Genes and Genomes analysis revealed that the functions of energy metabolism, signal transduction, and xenobiotics biodegradation and metabolism were increased in PxP-17, suggesting their potential roles in host adaptation. Metabolic profiling showed a significant difference in the abundance of gut metabolites between PxR and PxP-17, and significant correlations of gut bacteria with gut metabolites. These findings shed light on the interaction among plants, herbivores, and symbionts, and advance our understanding of host adaptation associated with gut bacteria and metabolic activities in *P. xylostella*.

## 1. Introduction

Bacterial symbionts are considered as “hidden players” in insect–plant interactions [1]. The digestive tract of a single insect can harbor a functionally complex and diverse microbial community [2]. Several studies have highlighted the multiple roles of microbiota in animal host physiology and fitness by modulating nutrient availability [3,4,5], protecting against biotic and abiotic stresses [6], and detoxifying plant secondary metabolites and soluble plant polysaccharides [7,8,9,10]. Moreover, gut microbes can be beneficial to herbivores in adaptation to a broad range of hosts [11,12,13]. For example, the gut microbes in *Probergrothius angolensis*, after the insect shifts from the host gymnosperm *Welwitschia* to the angiosperm Malvaceae, show a dramatic change that indicates that dynamic gut microbiota may help insects to rapidly adapt to new host plants [14].

Understanding the dynamic relationships between the structure of gut microbial communities and metabolites in insects during plant host shifts can contribute to a better understanding of herbivore ecology and improve the biocontrol of insect pests. However, studying gut metabolism and microbial symbionts in herbivores is complicated because of their changes in gene expression and consequently, of the resulting metabolic contents under varying environments [15,16]. Although it is possible to capture the substantial metabolic potential of gut microbiota in animals using high-throughput sequencing, precisely predicting the functional metabolic outputs of gut microbes from such data can be challenging. The identification and quantification of metabolites from host gut have recently become feasible with the advances in coverage and throughput analyses of metabolites [17,18]. Besides identifying the changes in gut microbial community in herbivores, especially in oligophagous or polyphagous herbivores that feed on different sources, the advent of metabolic profiling approaches provides a new scenario to the existing knowledge on gut ecosystems.

The Diamondback moth, *Plutella xylostella* (Lepidoptera: Plutellidae), is a destructive pest of a wide range of vegetable crops because of its rapid adaptability, high fecundity, insecticide resistance, short generation time, and capability to survive in different environments [19,20,21]. *P. xylostella* is mainly considered a specialist insect pest of Brassicaceae. However, Löhr et al. [22] reported a field population feeding on sugar snap pea, *Pisum sativum*, in Kenya. Under laboratory conditions, *P. xylostella* can rapidly adapt to peas, with larval survival rate increasing from 2.4% (first generation) to 49.7% (fourth generation). This adaptation to feeding on pea plants was identified as resulting from an autosomal oligogenic inheritance with “maternal effects” [23]. However, the role of microbial symbionts in *P. xylostella* adaptability to different host plants remains unknown.

We hypothesized that the shift in plant host may alter the insect’s gut microbiota and metabolites. Therefore, in the present study, we assessed the impact of different dietary sources on the gut microbial composition and metabolomic profile of *P. xylostella* using 16S rDNA sequencing and mass spectrometry-based metabolomics. Moreover, we described the gut microbial taxonomic composition in *P. xylostella* feeding on different host plants and predict each taxon’s metabolic function.

## 2. Results

### 2.1. Diversity of Gut Microbiota in P. xylostella Populations from Different Hosts

In total, 445,780 sequence reads were obtained from nine samples, of which 403,855 were used for further analysis after cleaning and trimming. The length of the sequence reads was of 200–540 bp, with 99.92% of them being of 400–440 bp. These sequence reads were clustered into 432 bacterial operational taxonomic units (OTUs).

**Figure 1:**
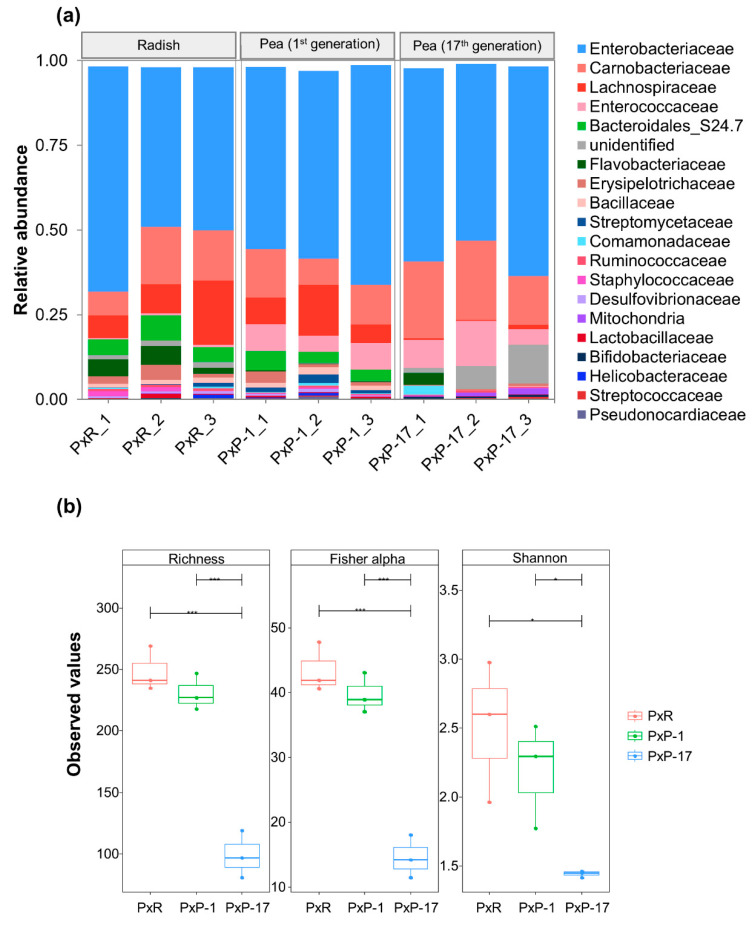
Gut microbial composition in different *P. xylostella* populations. (**a**) Relative abundance profiles at the family level; (**b**) Richness, Fisher’s alpha, and Shannon indices of gut microbiota in *P. xylostella*. Pair-wise ANOVA was performed between different treatments. “*” indicates *p* < 0.05; “***” indicates *p* < 0.001. PxR: *P. xylostella* feeding on radish; PxP-1 and PxP-17: the 1st and 17th generation of *P. xylostella*, respectively, feeding on peas after host shift from radish.

The rarefaction curves were flattened in all samples, indicating adequate sampling and successful retrieval of OTUs (Figure S1a). A total of 12 phyla, 24 classes, 46 orders, 76 families, and 165 genera were identified. The gut bacterial communities in all populations were dominated by the phyla Proteobacteria and Firmicutes (Figure S2). Among the top 20 bacterial families, Enterobacteriaceae was the dominant bacterial family across all samples (~50%), followed by Carnobacteriaceae (Figure 1a).

PxP-17 (17th generation of *P. xylostella* feeding on peas after host shift from radish) had a higher abundance of dominant bacteria but at a lower diversity compared to PxR (*P. xylostella* feeding on radish) and PxP-1 (1st generation of *P. xylostella* feeding on peas after host shift from radish) (Figure 1b and Figure S1b). The diversity of gut bacteria in PxP-1 was not significantly different from that of PxR, but those of PxR and PxP-1 were significantly higher than that of PxP-17 (Figure 1b).

**Figure 2:**
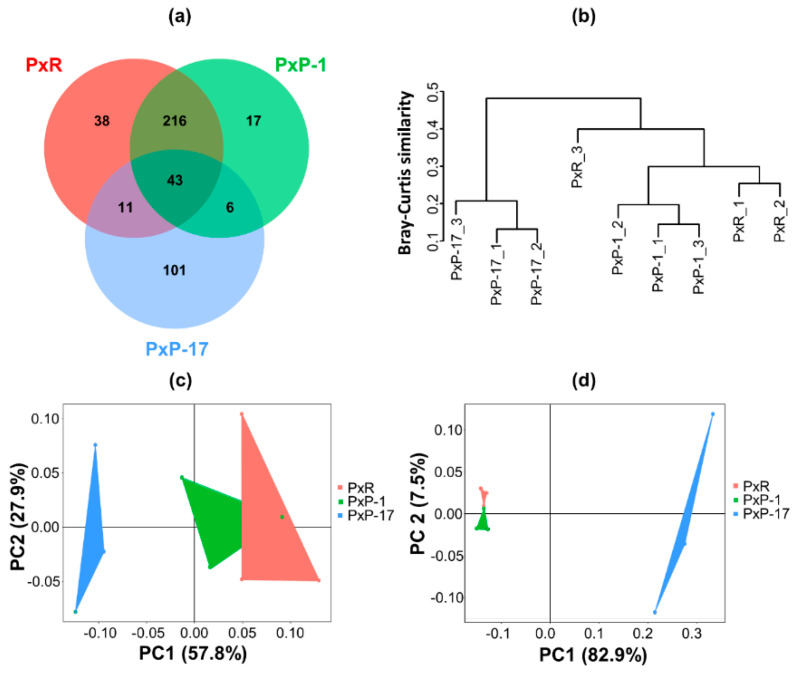
Diversity of the gut microbiota in different *P. xylostella* populations. (**a**) Venn diagram of operational taxonomic units (OTUs) in PxR, PxP-1, and PxP-17; (**b**) Cluster diagram including all samples based on Bray–Curtis distance dissimilarity; Principal Coordinate Analysis of (**c**) weighted UniFrac distances and (**d**) unweighted UniFrac distances, based on OTUs calculated at 97% similarity. PxR: *P. xylostella* feeding on radish; PxP-1 and PxP-17: the 1st and 17th generation of *P. xylostella*, respectively, feeding on peas after host shift from radish.

Only 43 OTUs were shared among the three populations. The gut microbiota of PxR and PxP-1 shared 259 of their 331 OTUs. However, the gut microbiota of PxP-17 differed from those of PxR and PxP-1 by having 101 unique OTUs among its 161 OTUs (Figure 2a). The cluster analysis and principal coordinate analysis (PCoA) ordination plot showed that gut bacterial composition in PxP-17 was significantly different from those of PxR and PxP-1 (Figure 2b–d; Table S1). The first two principal components of PCoA explained 85.7% and 90.4% of the total inertia based on the weighted (Figure 2c) and unweighted UniFrac distances (Figure 2d).

### 2.2. Abundance of Each Bacterial Family in the Gut Microbiota of P. xylostella Populations from Different Hosts

**Figure 3:**
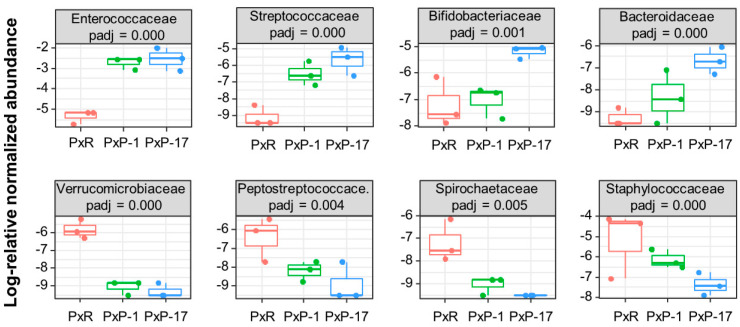
Trends of differential family abundance of the gut microbiota after host shift. Boxes show the interquartile ranges (25–75%), and the bands inside indicate median. Whiskers show the 1.5× upper or lower interquartile connected with a solid line. Pearson correlation test was performed. Padj corresponds to the *p*-value adjusted for multiple testing using the Benjamini–Hochberg method with a significance level of <0.01. PxR, *P. xylostella* feeding on radish; PxP-1 and PxP-17, the 1st and 17th generation of *P. xylostella*, respectively, feeding on peas after the host shift from radish.

The relative abundances of 26 out of 76 bacterial families were significantly different among PxR, PxP-1, and PxP-17. The abundance of Enterococcaceae, Streptococcaceae, Bifidobacteriaceae, and Bacteroidaceae showed a rising trend after host shift, whereas an opposite trend was observed for that of Verrucomicrobiaceae, Peptostreptococcace, Spirochaetaceae, and Staphylococcaceae (Figure 3).

Pasteurellaceae, Coriobacteriaceae, Veillonellaceae, Corynebacteriaceae, Fusobacteriaceae, and Clostridiales families XI and XIII were significantly more abundant in PxP-17 than in PxR and PxP-1, whereas the opposite trend was observed for Bacteroidales S-24.7, Lachnospiraceae, Desulfovibrionaceae, Bacillaceae, Streptomycetaceae, Xanthomonadaceae, Pseudonocardiaceae, Rikenellaceae, and Helicobacteraceae (Figure S3).

### 2.3. Functional Enrichment Profiles of Gut Microbes

**Figure 4:**
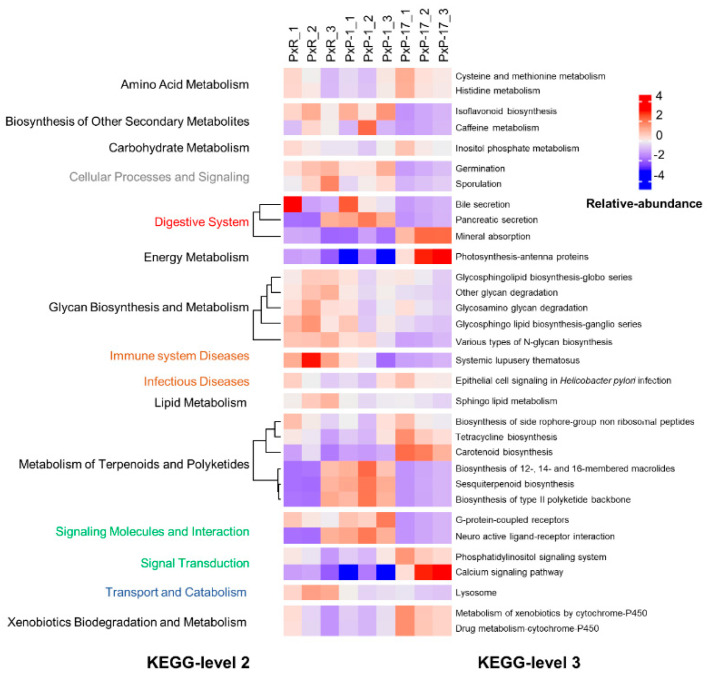
Gut microbiota functional profiles of each *P. xylostella* population at different Kyoto Encyclopedia of Genes and Genomes (KEGG) levels. PxR: *P. xylostella* feeding on radish; PxP-1 and PxP-17: the 1st and 17th generation of *P. xylostella*, respectively, feeding on peas after host shift from radish. Different colors in KEGG-level 2 represent different functions from those of KEGG-level 1, with black representing metabolism, grey representing unclassified function, red representing organismal systems, blue representing cellular processes, green representing environmental information processing, and orange representing human diseases.

The function of gut microbiota was predicted using the Kyoto Encyclopedia of Genes and Genomes (KEGG) levels 2 and 3 and clusters of orthologous groups (COGs) (Figure 4 and Figure S4). The roles of gut microbes in PxP-17 mostly comprised energy metabolism, signal transduction, xenobiotics biodegradation and metabolism, especially the calcium signaling pathway, and photosynthesis–antenna proteins. In contrast, roles such as biosynthesis of other secondary metabolites, cellular processes and signaling, glycan biosynthesis and metabolism, immune system diseases, lipid metabolism, signaling molecules and interaction, and transport and catabolism were reduced in PxP-17 (Figure 4). The COG functional enrichment analysis showed that PxP-1 contained more gut bacteria for encoding trehalose utilization protein (COG4813) than PxR, and that PxP-17 contained more peptidase m28 (COG2234), cytochrome C peroxidase (COG1858), and UPF0187 protein (COG3781) than PxR and PxP-1 (Figure S4 and Table S2). This indicates that these functional pathways may play important roles in dietary changes.

### 2.4. Profiling of the Midgut Metabolites in PxR and PxP-17

**Figure 5:**
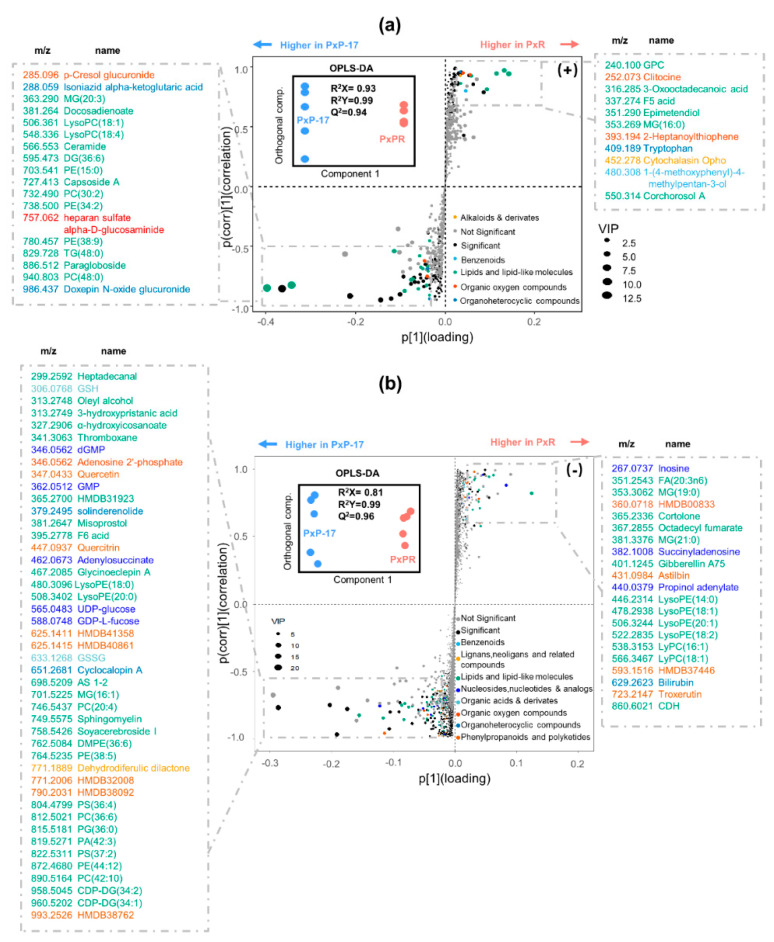
Comparison of metabolites between PxP-17 and PxR. (**a**) Positive mode (+); (**b**) negative mode (−). PxR: *P. xylostella* feeding on radish; PxP-17: the 17th generation of *P. xylostella* feeding on peas after host shift from radish. The inset box shows orthogonal projections to latent structures discriminant analysis (OPLS-DA) separation between PxP-17 and PxR along with the component that was used for correlating ion intensities. S-Plot obtained from OPLS-DA is shown in the main boxes. Each point represents accurate mass–retention time data. X axis is loadings; Y axis is correlation. Metabolites with absolute value of *p*(corr) > 0.5 are considered as significantly different in abundance between PxP-17 and PxR. The *m*/*z* [M + H]^+^ of the ions and annotations are shown in the dashed boxes with colors representing compounds from different classes.

Untargeted metabolomic profiling analyses identified several endogenous metabolites in larval midguts. A total of 1097 (+) and 5675 (−) ions were detected, and the abundance of 226 (+) and 1613 (−) ions were significantly different between PxP-17 and PxR (Figure S5). The orthogonal projections to latent structures discriminant analysis (OPLS-DA) of ion intensities revealed that PxR and PxP-17 were separated into two distinct clusters, indicating host-dependent metabolic profiles (Figure 5 and Table S3). In total, 62 (+) and 270 (−) differential ions contributed to the group differentiation (Figure S5), from which 30 (+) and 106 (−) ions were successfully annotated based on the human metabolome database (HMDB). In the S-plot, the significantly changed metabolites after host shift (absolute value of *p*(corr) > 0.5) were listed and classified into two groups containing nine categories (Figure 5). The majority of the annotated metabolites were lipids and lipid-like molecules (e.g., α-hydroxyicosanoate and 3-hydroxypristanic acid). Nucleosides (e.g., guanosine monophosphate (GMP), uridine diphosphate glucose (UDP-glucose), and GDP-L-fucose), organic acids (e.g., glutathione disulfide (GSSG) and glutathione (GSH)), organic oxygen compounds (e.g., adenosine 2′-phosphate), and phenylpropanoids and polyketides (flavonoids) were also detected (Figure 5). These results suggest that *P. xylostella* larvae feeding on different host plants show a clear divergence in midgut metabolomic profiling.

### 2.5. Associations between Gut Microbes and Metabolites in P. xylostella

**Figure 6:**
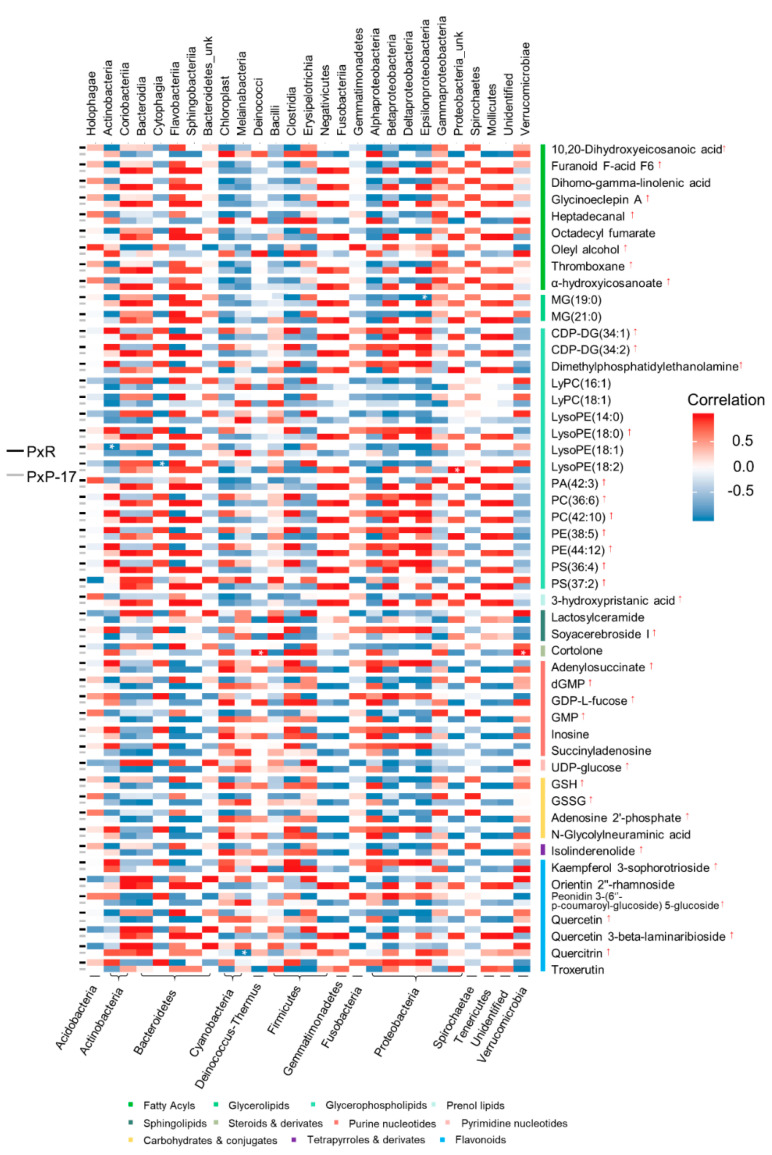
Relationships between gut bacteria classes and the top 50 most abundant metabolites in PxP-17 and PxR. Each bacterial class in the two populations was correlated with each metabolite. Asterisks represent statistical significance (*p* < 0.05). Red arrows indicate that the metabolite abundance was higher in PxP-17 than in PxR. PxR: *P. xylostella* feeding on radish; PxP-17: the 17th generation of *P. xylostella* feeding on peas after host shift from radish. MG: monoacylglycerols; CDP-DG: cytidine diphosphate diacylglycerol; PA: phosphatidic acid; PC: phosphatidylcholine; PE: phosphatidylethanolamine; PS: phosphatidylserine; LyPC: lysophosphatidylcholine; LysoPE: lysophosphatidylethanolamine.

Metabolites annotated using negative ionization and three closely clustered samples in OPLS-DA were tested for their correlations with bacterial communities because their sensitivity in compound detection was higher than that of metabolites with positive ionization. A heatmap showing the relationships among the top 50 gut compounds of PxR and PxP-17 and bacterial classes was generated (Figure 6). 

In PxR, Epsilonproteobacteria, Actinobacteria, and Cytophagia showed significant negative correlations with lipid molecules, including monoacylglycerols (MG) (19:0), lysophosphatidylcholine (LysoPE) (18:1), and LysoPE (18:2). In PxP-17, an unknown Proteobacteria class was significantly positively correlated with LysoPE (18:2). Bacteria from Deinococci and Verrucomicrobiae were significantly and positively correlated with cortolone (steroids), whereas Melainabacteria was significantly and negatively correlated with Quercitrin (flavonoids). Our results indicate that these bacteria-correlated metabolites belonged to lipid and xenobiotics, indicating that gut bacteria may participate in gut energy and detoxification metabolism.

## 3. Discussion

By exerting selective pressure to *P. xylostella* by providing only pea leaves as a food source under laboratory conditions, we successfully established a *P. xylostella* pea population. Moreover, analyzing gut microbiota and metabolites together, we found associations among host plant identity, gut microbiome composition, and gut metabolome for *P. xylostella*. Finally, we observed specific microbiome-intermediated correlations between host plants and metabolites. Taken together, our results suggest that gut microbiome composition may influence host metabolism and assist insects in adapting to a new host plant.

*P. xylostella* is currently one of the world’s most devastating pests because of its high adaptability [19]. It has, for instance, been reported to be resistant to almost all major classes of pesticides [20]. Besides, gut microbiota has been shown to contribute to its pesticide resistance, which suggests its putative importance in host adaptation [24]. *P. xylostella* was initially recognized as an oligophagous insect that fed only on Brassicaceae plants. However, Gupta and Thorsteinson [25] observed that *P. xylostella* could also use pea plants as a food source. Löhr and Gathu [22] further confirmed this observation based on *P. xylostella* feeding on peas in fields in Kenya and suggested the species’ capacity for host expansion and evolutionary adaptation.

Gut bacterial diversity in *P. xylostella* decreased after the host shift from radish to pea. Gut microbial diversity and relative gut microbial contents have been demonstrated to vary according to food type in insects such as *Bombyx mori* [26] and *Rothschildia lebeau* [27]. Cruciferous plants are the food source to which *P. xylostella* has been historically adapted. We found that the dominant bacteria in the gut content of *P. xylostella* from radish hosts were Firmicutes and Proteobacteria, which have been previously reported as the dominant bacteria in the intestines of *P. xylostella* larvae reared on crucifers, such as radish [28], Chinese cabbage, and cabbage [29]. Stably colonized gut bacteria, such as Proteobacteria, could be functionally crucial for insects to adapt to specific host plants, as previously demonstrated for pea aphids [30]. We also found that the gut microbiota in PxP-1 was similar to that in PxR, indicating that insects’ adaptation to new food resources is a gradual process. However, we did not monitor the continuous dynamic changes in generations of *P. xylostella* when adapting to a new plant host, which could help illuminate the key stage when the qualitative change occurred in the microbiota.

Microbial diversity significantly varied among the three populations, even at the family level, suggesting the insect’s adaptive plasticity to the host. The DESeq analysis also revealed significant changes in bacterial families. For example, compared with PxR, both PxP-1 and PxP-17 harbored abundant Enterococcaceae. Enterococcaceae can facilitate seed consumption in *Harpalus pensylvanicus* [31], reduce gut pH [32] and increase immunity [33] in *Bombyx mori*, provide nutrients for *Spodoptera littoralis* [34], and protect lepidopterans from *Bacillus thuringiensis* [35]. PxP-17 was associated with a decrease in Lachnospiraceae, which metabolize plant polysaccharides [36] and reduce graft-versus-host disease mortality [37]. Gut bacterial composition shifts qualitatively and quantitatively according to the host’s functional needs. The results indicate that the gut microbiota harbored in *P. xylostella* may co-evolve with the available food types and that it may participate in various metabolic functions in the host.

The OPLS-DA indicated a clear divergence in metabolic profile between PxR and PxP-17. Similarly, *Spodoptera frugiperda* feeding on different sources was shown to have different midgut metabolomic profiles [18]. Part of the differential compounds was derived from plant-based food. For example, we found that flavonoids (Quercetin and Kaempferol glycosides) were more abundant in PxP-17 than in PxR. Flavonoids, especially flavonols, can reduce the growth and survivorship of European corn borer, *Ostrinia nubilalis* [38]. Therefore, flavone glycosides may also restrain the larval development of *P. xylostella* feeding on peas. Organic acids, including GSH and GSSG, were accumulated in *P. xylostella* feeding on peas. GSH is an important antioxidant against toxic xenobiotics in living organisms and GSSG is a disulfide derived from two GSH molecules [39]. The high abundance of GSH and GSSG indicates that *P. xylostella* feeding on pea plants may face high oxidative stress. Future studies on this matter may help identify the differential metabolic pathways of *P. xylostella* feeding on different food resources. However, it is a limitation that we only compared the metabolic changes between PxR and PxP-17, which could only provide information regarding metabolic difference of the two populations. Continuous sampling from PxP-1 and subsequent generations can contribute to a better understanding of metabolite dynamics in the adaptation process.

The inter-omic analysis revealed correlations between microbial communities and metabolic profiles. Most bacteria-correlated metabolites have functions in lipid, amino acid, propionate, carbohydrate, or xenobiotics metabolism, highlighting the vital role of microbes in host energy and detoxification. On the one hand, microbes could contribute to nutrient intake. *Lactobacillus plantarum*, a commensal bacterium in *Drosophila*, can not only influence host energy uptake, but also promote host systemic growth by facilitating the insect host nutrient sensing system through controlling hormonal signals to enhance nutrient assimilation [40]. Further, microbes degrade secondary metabolites and convert secondary metabolites into nutrients. Gut microbiota in honeybee intestines degrades polymers in pollen and utilizes abundant pollen-derived substrates, e.g., flavonoids and outer pollen wall components, indicating a crucial role for degradation of recalcitrant secondary plant metabolites and pollen digestion [17,41]. In addition, *Eubacterium ramulus* and *Enterococcus casseliflavus* in the gut effectively degrade flavonoid compounds in foods, indicating the potential role of these bacteria in the transformation of flavonoids [42,43]. In our study, differential bacteria (e.g., Enterococcaceae) after host transfer may assist *P. xylostella* in degrading pea-derived secondary metabolites (e.g., flavonoids). Gut microbiota and metabolites are complex and often co-exist in the same host insects. Therefore, in many cases, we cannot determine the causal correlations between microbiota and metabolites, especially for less abundant but functionally important bacteria and metabolites. An alternative explanation may be that these correlations between gut metabolites and gut microbes are influenced by the nature of the host plant. Future studies isolating a single bacterium from a complex host gut microbiota are required. However, the isolated bacteria may not behave the same way as it does in a complex microbial community.

In conclusion, we used comparative microbiome and metabolome analyses to characterize the larval midgut of *P. xylostella* reared on pea and radish, and identified the bacteria and metabolites present in each population. The gut microbial diversity of *P. xylostella* feeding on a new and distant pea host was low compared to that of *P. xylostella* feeding on radish, indicating a change resulting from the adaptation to the new host. This study, to the best of our knowledge, is the first to characterize the gut metabolome of *P. xylostella* larvae and provide numerous candidate metabolites of *P. xylostella* feeding on Brassicaceae and Leguminosae. Gut bacterial communities are correlated with specific metabolites, suggesting that gut microbiota may assist in the adaptation to new host plants by regulating or participating in host metabolic processes. Our results improve our understanding of the interaction among plants, herbivores, and symbionts, and of host adaptation associated with gut bacteria and metabolic activities in herbivores, which could help unveil the mechanism of host adaptation or expansion in nature.

## 4. Materials and Methods 

### 4.1. Insect Rearing

**Figure 7:**
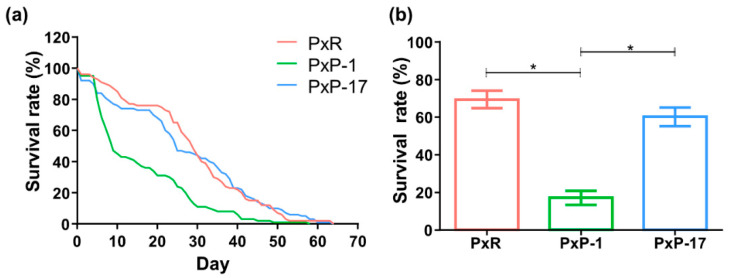
Survival rate of *P. xylostella* eggs to adults feeding on different host plants. (**a**) Accumulating survival curve; (**b**) survival rate. PxR: *P. xylostella* feeding on radish; PxP-1 and PxP-17: the 1st and 17th generation of *P. xylostella*, respectively, feeding on peas after host shift from radish. Student’s *t* test was performed between different treatments. “*” indicates *p* < 0.05.

*P. xylostella* specimens were initially collected from a cruciferous vegetable field in Fuzhou, China (26.08° N, 119.28° E) in July 2004 and then reared on radish (*Raphanus sativus*) in the laboratory [44]; this population is hereafter referred to as PxR. In January 2018, *P. xylostella* eggs from the PxR population were artificially placed on pea (*Pisum sativum*) leaves to establish a new population in the laboratory (hereafter referred to as PxP). In the 1st generation of *P. xylostella* after host shift (hereafter referred to as PxP-1), few individuals completed their life cycle and the survival rate was low (<20%). In the 17th generation after host shift (hereafter referred to as PxP-17), the survival rate increased to 60%, showing no significant difference from that of PxR individuals (Figure 7). Individuals from the PxP-1 and PxP-17 populations were thus collected and compared. Both *P. xylostella* populations were kept in a controlled-environment chamber (23 ± 1 °C, 65 ± 5% relative humidity, and 16 light: 8 dark photoperiod). Adults were supplied with 10% honey solution.

### 4.2. Sample Collection

For 16S rDNA sequencing, gut contents from 30 4th-instar larvae from each treatment (PxR, PxP-1 and PxP-17) were used. For this, larvae were first treated with 75% ethanol for 90 s, rinsed three times, and then dissected in sterile 1% phosphate-buffered saline (PBS) under the microscope. Gut contents were supplemented with 1 mL 1% PBS, temporarily frozen in liquid nitrogen, and eventually stored at −80 °C. Three replicates per treatment were collected.

For metabolome analysis, only the metabolic difference of PxR and PxP-17 were compared, as PxP-1 had a low survival rate and its microbial structure showed no significant difference from that of PxR. The midguts of 30 4th-instar larvae from each population were analyzed as a replicate. For this, larvae were surface sterilized as described above and dissected. Then, gut contents were removed so that those clean midguts could be obtained. Midguts were temporarily frozen in liquid nitrogen and eventually stored at −80 °C. Five replicates from each of the two populations were obtained.

### 4.3. 16S rDNA Sequencing

Bacterial DNA was extracted from the gut content samples using the TIANamp Bacteria DNA Kit (TIANGEN BIOTECH, Beijing, China). The final quantity and quality of the extracted DNA were evaluated on 1% agarose gel and a NanoDrop 2000 spectrophotometer (Thermo Fisher Scientific, Wilmington, DE, USA). The pair of primers 336F (5′-GTACTCCTACGGGAGGCAGCA-3′) and 806R (5′-GTGGACTACHVGGGTWTCTAAT-3′) were used to amplify the V3-V4 hypervariable region of the bacterial 16S rRNA gene. For each sample, a 10-digit barcode sequence (provided by Allwegene Company, Beijing, China) was added to the 5′ end of the forward and reverse primers. Polymerase chain reaction (PCR) was performed in a 25 µL reaction volume containing 30 ng of genomic DNA, 1 µL of each barcoded primer (5 µM), 3 µL of albumin from bovine serum (BSA) (2 ng/µL), 12.5 µL of 2× Taq Plus Master Mix, and appropriate ddH_2_O. Cycling parameters were 94 °C for 5 min, followed by 25 cycles at 94 °C for 30 s, 50 °C for 30 s, and 72 °C for 60 s, and a final extension at 72 °C for 7 min. The PCR products were purified from 1.0% agarose gels and quantified using the Qubit fluorescence quantitative system. Paired-end sequencing was performed on the Illumina platform MiSeqPE 300 (Allwegene Technology Co., Ltd., Beijing, China).

Sequences were grouped into OTUs at a 97% similarity level using UPARSE [45]. The ribosomal database project (RDP) classifier (Version 16) was used to assign sequences to different taxonomic groups based on the Greengene ribosomal RNA gene database (Version 13.5) [46]. The sequences were submitted to the Sequence Read Archive in National Center for Biotechnology Information (NCBI, https://www.ncbi.nlm.nih.gov/; accession numbers SRR11206458–SRR11206466).

### 4.4. Metabolite Extraction and Profiling

Metabolites were extracted from the gut of 4th-instar *P. xylostella* larvae as previously described [47] with minor modifications. Samples were homogenized with 300 µL of prechilled methanol/water (1:1) using a TissueLyser (Qiagen, Hilden, Germany) at 25 Hz for 3 min. Homogenized mixtures were centrifuged at 4 °C and 12,000 rpm for 10 min to remove proteins and debris. Supernatants were transferred into Eppendorf tubes and evaporated by a Savant vacuum concentrator (Eppendorf, Germany) at 45 °C for 60 min. Remaining solutes were re-dissolved using 200 µL of prechilled methanol/water (1:1), filtered through 0.22-µmol Millipore Filters (Waters, Milford, MA, USA), and stored in Chromatographic bottles.

Liquid chromatograph–mass spectrometry (LC–MS) was performed on an ACQUITY Ultra Performance Liquid Chromatography (UPLC) system coupled to a Vion ion mobility spectrometry (IMS) Q time-of-flight (Tof) mass spectrometer (Waters Corporation, USA). The separation of all samples was performed on an ACQUITY UPLC BEH C18 column (2.1 × 100 mm, 1.7 µm particle size; Waters Corporation). Solvent A (0.1% formic acid aqueous solution) and solvent B (0.1% formic acid-acetonitrile solution) were used for gradient elution. The gradient program was as follows: 0–2 min 99.9% A; 2–6 min 75% A; 6–10 min 20% A; 10–12 min 10% A; 12–21 min 0.1% A; 21–23 min 0.1% A; 23–24 min, 99.9% A; 24–26 min 99.9% A. Sample injection volume was 5 µL, and flow rate was 0.3 mL/min. Sample temperature was 4 °C, and column temperature was 40 °C. Both positive (+) and negative (−) modes were used for the detection of compounds in mass spectrometry. The full-scan range was 50–1000 *m*/*z*. Electrospray ionization conditions were as follows: capillary voltage 3 kV (+) or 2.5 kV (−), source temperature 120 °C, desolvation temperature 450 °C, cone gas flow 25 L/h, desolvation gas flow 900 L/h, low collision energy 6 eV, and high collision energy 30 to 50 eV.

Progenesis QI (Nonlinear Dynamics, waters) was used to visualize the LC–MS results. Three stringent conditions were set to filter analytes: an analysis of variance (ANOVA) *p*-value ≤ 0.05, a fold change (FC) ≥ 2, and a minimum coefficient of variation (CV) ≤ 30, which could reduce the “false discovery rate”. The chosen compounds were exported to Ezinfo (Version 3.0, Umetrics, Umea, Sweden) for analysis. OPLS-DA was conducted to sort ion changes based on the differences in the explanation capabilities of the PxR and PxP-17 metabolome profiles [48]. Ezinfo 3.0 was also used to filter compounds and metabolites with high variable importance in projection (VIP > 1) scores contributing to group differentiations in the OPLS-DA plot [49,50]. Differentiated filtered ions were re-imported into Progenesis QI for identification using the HMDB (http://www.hmdb.ca/) and Metlin database (http://metlin.scripps.edu). Data were deposited in the European bioinformatics institute of the European molecular biology laboratory (EMBL-EBI) under accession code MTBLS1743 (http://www.ebi.ac.uk/metabolights/MTBLS1743) and will be accessible upon publication of the manuscript [51].

### 4.5. Data Analyses

All analyses were performed in R (Version 3.5.2, Vienna, Austria) [52]. Rarefaction and rank–abundance curves were generated using the ”Biodiversity”package [53]. Taxonomic composition plots were made using the “phyloseq” package [54]. Alpha diversity analysis of microbial abundance data was performed using the “microbiomeSeq” package [55]. Permutational multivariate analysis of variance (PERMANOVA) and PCoA ordination plot were performed based on UniFrac distances (weighted and unweighted) and Bray–Curtis dissimilarity matrices using the packages “phangorn” [56] and “pairwiseAdonis” [57]. Differential sequence abundance analysis was conducted using the “DESeq2” package [58]. Prediction of microbial function was performed using phylogenetic investigation of communities by reconstruction of unobserved states (PICRUSt, Version 1.1.4, https://picrust.github.io/picrust/install.html). Obtained OTUs were normalized by 16S rRNA copy numbers. Functional genes were predicted using the KEGG (http://www.genome.jp/kegg/) catalog and evolutionary genealogy of genes: non-supervised orthologous groups database (EggNOG; http://eggnog.embl.de/).

The comparison of metabolites between *P. xylostella* from radish and pea (S-plot) was performed using the “ggplot2” package [59]. A microbe–metabolite correlation heatmap was created by calculating Pearson correlation coefficients for each pairwise combination of microbial taxa abundances and metabolite intensities using the “ComplexHeatmap” and “circlize” packages [60]. Furthermore, *p*-values were adjusted using the Benjamini–Hochberg method with a significance level of <0.01 for multiple correlation testing.

## Supplementary Materials

The Supplementary Materials can be found at https://www.mdpi.com/1422-0067/21/17/6283/s1. Figure S1: Distribution of operational taxonomic units (OTUs) in PxR, PxP-1, and PxP-17. Figure S2: Relative abundance of gut bacteria at different taxonomic levels. Figure S3: Fluctuations of differential family abundance of the gut microbiota after host shift. Figure S4: Clusters of orthologous group (COG) functional profiles of gut microbiota in PxR, PxP-1, and PxP-17. Figure S5: Differentially accumulated metabolites in PxP-17 and PxR. Table S1: Differences in the bacterial operational taxonomic units (OTUs) community structure between *P. xylostella* populations on different host plants. Tables S2 and S3: Supporting data sets.
