# Supplementary material for: Differential Profiles of Gut Microbiota and Metabolites Associated with Host Shift of Plutella xylostella
Source: Int J Mol Sci. 2020 Aug 30;21(17):6283. doi: 10.3390/ijms21176283 (PMC7504026; doi:10.3390/ijms21176283)
Supplement: Supplementary file 1 [file ijms-21-06283-s001.zip › Supporting information.doc]

**Table S1.** **Differences in the bacterial operational taxonomic units (OTUs) community structure between**

***P. xylostella* populations on different host plants.**

| **Distance metrices** | **df** | **SS** | **MS** | ***F*-value** | **R^2^** | ***p*-value** |
| --- | --- | --- | --- | --- | --- | --- |
| Weighted unifrac | 2 | 0.040 | 0.020 | 3.279 | 0.522 | 0.028^*^ |
| Unweighted unifrac | 2 | 0.356 | 0.178 | 16.920 | 0.849 | 0.006^**^ |
| Bray-Curtis distance | 2 | 0.210 | 0.105 | 3.862 | 0.563 | 0.008^**^ |

Permutational multivariate analysis of variance analysis (PERMANOVA) was used. Df, degrees of freedom; SS, sum of squares; MS, mean sum of squares; R^2^, % variation explained; *F*-value, *F* value by permutation. Asterisks indicate the degree of significance (* indicates *p* < 0.05; ** *p* < 0.01). The *p*-values are based on 999 permutations.

**Figure S1. Distribution of operational taxonomic units (OTUs) in PxR, PxP-1, and PxP-17.** (a) Rarefaction curves; (b) Rank-abundance curves. PxR: *P. xylostella* feeding on radish; PxP-1 and PxP-17: the 1^st^ and 17^th^ generation of *P. xylostella*, respectively, feeding on peas after host shift from radish.

**Figure S2. Relative abundance of gut bacteria at different taxonomic levels.** (a) phylum; (b) class; (c) order; (d) genus. PxR: *P. xylostella* feeding on radish; PxP-1 and PxP-17: the 1^st^ and 17^th^ generation of *P. xylostella*, respectively, feeding on peas after host shift from radish.

**Figure S3. Fluctuations of differential family abundance of the gut microbiota after host shift.** The box shows the interquartile range (25-75%) and the band inside indicates the median. Whiskers show the 1.5 times of the upper or lower interquartile connected with solid line. Padj, corresponded to *p*-value adjusted for multiple testing using Benjamini-Hochberg method with a significance level of <0.01. PxR, *P. xylostella* feeding on radish, PxP-1 and PxP-17, the 1^st^ and later generation of *P. xylostella* feeding on pea after the host shift from radish.

**Figure S4. Clusters of orthologous group (COG) functional profiles of gut microbiota in PxR, PxP-1, and PxP-17.** PxR: *P. xylostella* feeding on radish; PxP-1 and PxP-17: the 1^st^ and 17^th^ generation of *P. xylostella*, respectively, feeding on peas after host shift from radish.

**Figure S5. Differentially accumulated metabolites in PxP-17 and PxR**. (a) Positive detected mode (+); (b) Negative detected mode (-). Black triangle indicates that the metabolites were filtered by *p*-value ≤ 0.05, fold Change (FC) ≥ 2, and variable importance in projection (VIP) > 1. PxR: *P. xylostella* feeding on radish; PxP-17: the 17^th^ generation of *P. xylostella* feeding on peas after host shift from radish.
